# Digital Health as an Enabler of Personalized Medicine in Ghana: Gaps, Opportunities, and Future Directions

**DOI:** 10.2196/70696

**Published:** 2026-04-16

**Authors:** Godsway Sackey, Babajide Owoyele, Frank E Baiden, Stefan Konigorski

**Affiliations:** 1 Hasso Plattner Institute for Digital Engineering Potsdam, Brandenburg Germany; 2 Digital Engineering Faculty University of Potsdam Potsdam, 14482 Germany; 3 Fred N Binka School of Public Health, University of Health and Allied Sciences Ho Ghana; 4 Hasso Plattner Institute for Digital Health at Mount Sinai Icahn School of Medicine at Mount Sinai New York, NY United States

**Keywords:** personalized medicine, digital health, health interventions, Ghana, N-of-1 trials

## Abstract

Digital health solutions and personalized medicine are increasingly promoted as pathways to improve health care delivery in low-resource settings, including Ghana. Drawing on insights from our examination of the published literature and our engagement with digital health research in this context, we present a scholarly viewpoint on how digital health has been positioned in relation to personalized medicine in Ghana, where progress has been uneven and largely oriented toward population-level interventions. We observe that most digital health initiatives in Ghana focus on mobile health apps and health information systems that support service delivery and access, with limited translation toward truly personalized models of care. Although personalized medicine is frequently discussed as a future goal, it remains weakly operationalized in practice, and approaches such as N-of-1 trials—often cited as exemplars of individualized care—are notably absent from the existing literature. Importantly, the limited uptake of personalized approaches does not reflect a lack of relevance in Ghana, but rather the constraints of population-level digital health strategies that, while essential, have shown limited capacity to address individual heterogeneity in treatment responses, adherence, and long-term outcomes. We argue that this absence reflects structural, methodological, and policy-related challenges. At the same time, emerging digital health infrastructure, policy interest, and research capacity present opportunities to reposition digital health as an enabler of personalized medicine. Helping many single individuals through scalable digital personalized approaches may be a valuable innovative approach to public health. This viewpoint articulates key gaps, contextual constraints, and future directions, with the aim of informing researchers, policymakers, and implementers seeking to advance personalized, data-driven care in Ghana and comparable settings.

## Reframing Digital Health and Personalized Medicine in Ghana

Digital health has become a central pillar of health system strengthening efforts in many low- and middle-income countries, including Ghana, where mobile health apps, electronic health records, and digital decision-support tools are increasingly integrated into service delivery [1-3]. These initiatives are commonly framed around improving access, efficiency, and continuity of care, particularly in settings constrained by limited infrastructure and health workforce capacity [4-6]. However, considerably less attention has been given to how digital health might meaningfully enable personalized models of care that respond to individual patient needs rather than population averages [6-9].

Personalized medicine, broadly understood as the tailoring of prevention, diagnosis, and treatment strategies to individual characteristics, has gained prominence in high-income settings but remains conceptually and operationally underdeveloped in many low-resource contexts. In Ghana, discussions of personalized medicine are often aspirational, with limited clarity on how existing digital health systems could support individualized decision-making in routine practice [3,10]. As a result, digital health and personalized medicine are frequently discussed in parallel rather than as mutually reinforcing domains [11-13].

Our examination of the published literature on digital health in Ghana suggests that most existing initiatives prioritize generalized service delivery improvements, such as appointment reminders, health education, surveillance, and data management [3,14,15]. While these efforts are important, they rarely extend toward designs that support individual-level experimentation, adaptive treatment strategies, or sustained personalization over time. Notably, approaches such as N-of-1 trials—which are single-person crossover trials that are often highlighted in the personalized medicine discourse as a rigorous method for individual-level evaluation—are absent from the Ghanaian digital health literature. We contend that this absence reflects structural, methodological, and policy-related barriers rather than a lack of relevance or potential [5,10,16].

This viewpoint argues that reframing digital health as a potential enabler of personalized medicine in Ghana requires greater conceptual precision, contextual sensitivity, and deliberate alignment between technological design, research methods, and health system priorities. Rather than treating the current evidence base as a deficit, we use it as a foundation to articulate key gaps, clarify misconceptions, and outline future directions for research, policy, and implementation.

## What the Existing Evidence Suggests About Digital Health and Personalized Medicine in Ghana

Our perspective is informed by a protocol-guided examination of the published literature on digital health, personalized medicine, and N-of-1 trials in Ghana. This examination followed a preregistered protocol to ensure transparency and methodological rigor, with the protocol publicly available through the Open Science Framework [17]. We systematically searched multiple bibliographic databases, including PubMed, Scopus, IEEE Xplore, the Cochrane Library, and Google Scholar, covering the period from 2000 to 2024. Retrieved records were screened and charted using predefined criteria to map intervention types, thematic focus, and the extent to which individualized approaches were operationalized in practice. This examination revealed several consistent patterns relevant to the advancement of individualized care.

First, the existing body of work is relatively small—in total, 40 relevant studies were identified across the entire search period—and unevenly distributed, with most studies concentrated on digital health interventions designed to improve access, efficiency, and service delivery at the population level. Mobile health apps and electronic health record systems dominate this landscape, reflecting national and institutional priorities around digitization and health system strengthening [6,7,18]. More specifically, mobile health interventions constituted approximately 60% of identified studies, followed by electronic health record and broader eHealth system implementations (approximately one quarter of studies). Explicitly personalized medicine applications accounted for less than 10% of publications, and no empirical applications of N-of-1 trials were identified. This distribution illustrates the strong orientation toward population-level digital service delivery, with comparatively limited operationalization of individualized care models.

Second, personalized medicine remains weakly operationalized in practice within Ghana and possibly also other low-resource settings, despite being frequently invoked in conceptual discussions. Only a small subset of the literature engages explicitly with personalization beyond stratified or protocol-driven approaches, and even fewer studies articulate how individual-level data could be used to iteratively adapt care over time. This pattern suggests that personalization is often treated as a future aspiration rather than a design principle embedded in current digital health initiatives [11,12].

Third, we did not identify any empirical applications of N-of-1 trials within the Ghanaian digital health literature, despite the theoretical alignment between digital health and individualized experimentation and despite frequent conceptual discussion of personalized medicine in the literature. N-of-1 trials are widely described in the personalized medicine literature as a rigorous approach to evaluating treatment effects at the individual level, particularly for chronic and heterogeneous conditions [19-21]. We contend that their absence in the Ghanaian context reflects structural, methodological, and regulatory barriers rather than a lack of relevance or potential.

Finally, the Ghana-based studies within the reviewed literature reflect both national investment in digital health and the way research agendas are shaped by funding priorities, policy emphasis, and data availability. While insights from comparable low-resource settings are informative, the Ghanaian case illustrates how contextual factors—such as governance structures, digital infrastructure, and workforce capacity—mediate the translation of digital health tools into more personalized models of care [22-24]. For instance, assessments of Ghana’s eHealth workforce reveal that limited training in health informatics restricts the ability of frontline workers to utilize digital tools for complex, adaptive decision-making beyond basic data entry. Similarly, the fragmentation of electronic health record systems often prevents the interoperability needed to generate the longitudinal patient profiles essential for personalized care [3,5]. Taken together, these observations provide a foundation for rethinking how digital health initiatives might evolve beyond generalized interventions toward approaches that better support individual-level decision-making.

Although this analysis is grounded in evidence from Ghana, several of the patterns identified are likely to extend to other low- and middle-income settings pursuing digital health-enabled health system strengthening. Across studies in Ghana and comparable low- and middle-income countries, digital health interventions are frequently designed as fixed, protocol-driven tools aimed at standardized service delivery, often embedded within predefined reporting and administrative workflows [6,7,25]. Although appropriate for scaling essential services, such designs rarely incorporate mechanisms for systematic within-person learning or adaptive treatment refinement over time. While these approaches have delivered important gains, they have also revealed shared limitations in addressing individual heterogeneity in disease trajectories, treatment response, and long-term care needs, particularly as health systems face a growing burden of chronic and non-communicable conditions [26,27]. For example, mobile health interventions for noncommunicable diseases often rely on standardized SMS text messaging protocols that do not dynamically adapt to a patient’s specific adherence barriers or changing health status [26]. Similarly, clinical decision support systems used in maternal care are frequently optimized for strict guideline adherence, often missing the unique, evolving risk profiles of individual patients [28].

The Ghanaian case thus offers broader insight into how digital health investments, when primarily oriented toward population-level objectives, may inadvertently constrain the development of more individualized models of care. Challenges related to workforce capacity, data governance, and the use of routinely collected digital data for adaptive decision-making recur across many resource-constrained settings. At the same time, Ghana’s relatively mature digital health ecosystem illustrates how existing infrastructure could be leveraged to support more personalized approaches without requiring fundamentally new systems. These insights may therefore inform efforts in other settings seeking to move beyond generalized digital health interventions toward more personalized, data-driven care.

## Implications for Research, Policy, and Practice

The patterns observed in the existing literature have important implications for how digital health and personalized medicine are conceptualized and pursued in Ghana. First, there is a need to move beyond viewing digital health solely as a mechanism for improving access and efficiency toward recognizing its potential to support adaptive, patient-centered care. This shift requires greater attention to how data generated through routine digital health interventions can be leveraged to inform individual-level decision-making over time, rather than serving primarily administrative or surveillance functions [25,29].

Beyond methodological considerations, the existing literature points to a set of recurring system-level constraints that limit the translation of digital health into more personalized models of care in Ghana. Across studies, digital health interventions are frequently designed as fixed, protocol-driven tools aimed at standardized service delivery, with limited flexibility for individual-level adaptation over time [14,28,30]. Data generated through these systems are often used primarily for reporting, surveillance, or administrative purposes, rather than as inputs for ongoing clinical decision-making at the individual level. This design orientation constrains the ability of digital platforms to support learning about what works for whom, even when longitudinal data are technically available.

In addition, workforce capacity, data governance arrangements, and regulatory uncertainty emerge as cross-cutting barriers. Health workers are rarely trained to interpret or act on individual-level digital data beyond predefined workflows, and guidance on secondary data use, experimentation, and adaptive care remains limited. These constraints are not unique to Ghana, but they are amplified in resource-constrained settings where incentives favor scale and coverage over experimentation and iteration. As a result, even well-established digital health systems may inadvertently reinforce standardized care pathways rather than enabling personalization.

Importantly, these barriers help explain the absence of approaches such as N-of-1 trials in the Ghanaian literature. Their lack of uptake reflects structural and institutional conditions rather than conceptual misalignment or lack of potential. In particular, the rigid architecture of current electronic health record systems is primarily designed for aggregate reporting to national databases, making it difficult to integrate the granular, high-frequency patient feedback loops that N-of-1 trials require [2,3]. Addressing these constraints—through targeted capacity building, clearer governance frameworks, and deliberate design choices—may therefore be as important as introducing new technologies when seeking to advance personalized, data-driven care.

From a research perspective, advancing personalized medicine in Ghana will require methodological innovation that is sensitive to local constraints. While randomized controlled trials remain important, alternative designs such as pragmatic trials, adaptive studies, and N-of-1 trials may offer more feasible and contextually appropriate pathways for evaluating individualized interventions in resource-constrained settings [19,20]. Building capacity in these methods, alongside strengthening data governance and ethical oversight, will be critical to ensuring rigor and trust. Calls for innovative evidence-generation approaches in digital health emphasize the need for pragmatic and adaptive evaluation models that reflect real-world implementation constraints rather than relying exclusively on traditional randomized controlled trials [6]. In resource-constrained settings, digitally supported N-of-1 designs using open-source platforms such as the StudyU platform [21] may represent one such approach, offering methodological rigor while remaining scalable and embedded within routine service delivery. Aligning digital infrastructure with adaptive research designs could therefore bridge the current gap between population-scale digital health implementation and genuinely individualized care.

Global analyses of digital health ethics and governance similarly emphasize that regulatory frameworks often prioritize data protection, reporting compliance and system interoperability rather than enabling responsible secondary use of routinely collected data for adaptive care or individual-level experimentation [29,31]. Without clearer guidance on permissible experimental designs within routine care settings, digital platforms risk remaining administratively efficient but clinically static. In Ghana, ongoing digital health and health information system reforms provide an opportunity to embed personalization principles into national strategies rather than treating them as add-on innovations [6,31].

Finally, implementers and health system leaders must grapple with the practical realities of integrating personalized approaches into routine care. This includes aligning digital tools with clinical workflows, supporting health workers through training and decision support, and ensuring that personalization efforts do not exacerbate existing inequities. Without deliberate attention to these issues, digital health risks reinforcing standardized, one-size-fits-all models of care, even as it expands technological capacity [24].

## Concluding Perspective

A recurring critique of personalized approaches in low-resource settings, including Ghana, is that they appear misaligned with urgent population-level public health needs. This concern is understandable, given the central role of population-based strategies in expanding coverage, improving equity, and strengthening health systems. However, Ghana’s experience with digital health over the past decade suggests that population-level approaches alone—while necessary—have not been sufficient to address persistent challenges related to individual variability in disease trajectories, treatment response, adherence, and long-term care, particularly for chronic and noncommunicable conditions. Many digital health interventions remain designed around standardized protocols and average effects, which can obscure unmet needs at the individual level even when population-level gains are achieved.

In this context, personalized approaches should not be viewed as competing with public health priorities but as a complementary evolution enabled by digital health. By leveraging routinely collected individual-level data, scalable digital personalized approaches offer a pathway to improve effectiveness without sacrificing reach or equity. Helping many single individuals through such scalable personalized strategies may therefore represent a pragmatic extension of public health practice in Ghana rather than a departure from it.

Digital health has already transformed aspects of health care delivery in Ghana [3,32], yet its potential to support personalized medicine remains largely unrealized. By reframing existing digital health investments as platforms for individualized, adaptive care—and by addressing the methodological, policy, and system-level barriers that currently constrain personalization—there is an opportunity to move beyond incremental gains toward more patient-centered models of care. This viewpoint calls for a deliberate shift in how digital health is conceptualized, evaluated, and implemented, positioning personalized medicine not as a distant aspiration but as an achievable goal within Ghana’s evolving health system.

In this setting, N-of-1 trials provide a practical illustration of how personalized approaches could be implemented through digital health without undermining population-level priorities. Although such designs have not yet been applied in Ghana, their absence reflects structural, methodological, and system-level constraints rather than limited relevance. Examining whether and how digitally supported N-of-1 trials could be adapted to Ghana’s health system represents a logical next step for research and implementation.

## Data Availability

The data underlying this study consist of bibliographic records identified through systematic database searches covering the period from January 2000 to April 2024. The full search strategy is provided in Multimedia Appendix 1. Extracted study characteristics and the cleaned integrated dataset generated during the literature examination are available from the corresponding author upon request. No individual-level participant data were generated or analyzed in this study.

## Appendix

Multimedia Appendix 1 Search strategy and protocol details.

## References

[1] Amoakoh HB, Klipstein-Grobusch K, Amoakoh-Coleman M, Agyepong IA, Kayode GA, Sarpong C, Grobbee DE, Ansah EK (2017). The effect of a clinical decision-making mHealth support system on maternal and neonatal mortality and morbidity in Ghana: study protocol for a cluster randomized controlled trial. Trials.

[2] Lee S, Lee YJ, Kim S, Choi W, Jeong Y, Rhim NJ, Seo I, Kim SY (2022). Perceptions on data quality, use, and management following the adoption of tablet-based electronic health records: results from a pre–post survey with district health officers in Ghana. JMDH.

[3] Achampong EK (2022). Implementation of electronic health record system in Ghana: a review. TOPHJ.

[4] Long LA, Pariyo G, Kallander K (2018). Digital technologies for health workforce development in low- and middle-income countries: a scoping review. Glob Health Sci Pract.

[5] Ogoe HA, Asamani JA, Hochheiser H, Douglas GP (2018). Assessing Ghana's eHealth workforce: implications for planning and training. Hum Resour Health.

[6] Labrique AB, Wadhwani C, Williams KA, Lamptey P, Hesp C, Luk R, Aerts A (2018). Best practices in scaling digital health in low and middle income countries. Global Health.

[7] Karamagi HC, Muneene D, Droti B, Jepchumba V, Okeibunor JC, Nabyonga J, Asamani JA, Traore M, Kipruto H (2022). eHealth or e-Chaos: The use of digital health interventions for health systems strengthening in Sub-Saharan Africa over the last 10 years: a scoping review. J Glob Health.

[8] Fatehi F, Samadbeik M, Kazemi A (2020). What is digital health? Review of definitions. Stud Health Technol Inform.

[9] Holeman I, Cookson T, Pagliari C (2016). Digital technology for health sector governance in low-and middle-income countries. J Glob Health.

[10] Kesse-Tachi A, Asmah AE, Agbozo E (2019). Factors influencing adoption of eHealth technologies in Ghana. Digit Health.

[11] Mathur S, Sutton J (2017). Personalized medicine could transform healthcare. Biomed Rep.

[12] Shabaruddin FH, Fleeman ND, Payne K (2015). Economic evaluations of personalized medicine: existing challenges and current developments. Pharmgenomics Pers Med.

[13] Mitropoulos K, Cooper DN, Mitropoulou C, Agathos S, Reichardt JKV, Al-Maskari F, Chantratita W, Wonkam A, Dandara C, Katsila T, Lopez-Correa C, Ali BR, Patrinos GP (2017). Genomic medicine without borders: which strategies should developing countries employ to invest in precision medicine? A new "fast-second winner" strategy. OMICS.

[14] Mohammed A, Acheampong PR, Otupiri E, Osei FA, Larson-Reindorf R, Owusu-Dabo E (2019). Mobile phone short message service (SMS) as a malaria control tool: a quasi-experimental study. BMC Public Health.

[15] Rokicki S, Fink G (2017). Assessing the reach and effectiveness of mHealth: evidence from a reproductive health program for adolescent girls in Ghana. BMC Public Health.

[16] Barkman C, Weinehall L (2017). Policymakers and mHealth: roles and expectations, with observations from Ethiopia, Ghana and Sweden. Glob Health Action.

[17] Exploring digital health solutions: personalised medicine and n-of-1 trials in Ghana: a scoping review. Open Science Framework.

[18] Abul-Husn NS, Kenny EE (2019). Personalized medicine and the power of electronic health records. Cell.

[19] Lillie E, Patay B, Diamant J, Issell B, Topol EJ, Schork NJ (2011). The n-of-1 clinical trial: the ultimate strategy for individualizing medicine?. Per Med.

[20] Alemayehu C, Nikles J, Mitchell G (2018). N-of-1 trials in the clinical care of patients in developing countries: a systematic review. Trials.

[21] Konigorski S, Wernicke S, Slosarek T, Zenner A, Strelow N, Ruether DF, Henschel F, Manaswini M, Pottbäcker Fabian, Edelman JA, Owoyele B, Danieletto M, Golden E, Zweig M, Nadkarni GN, Böttinger Erwin (2022). StudyU: a platform for designing and conducting innovative digital N-of-1 trials. J Med Internet Res.

[22] Dzando G, Akpeke H, Kumah A, Agada E, Lartey AA, Nortu J, Nutakor HS, Donyi AB, Dordunu R (2022). Telemedicine in Ghana: insight into the past and present, a narrative review of literature amidst the Coronavirus pandemic. J Public Health Afr.

[23] Koduah A, Anim Boadi Jessica, Azeez JNK, Adu Asare Brian, Yevutsey S, Gyansa-Lutterodt M, Nonvignon J (2023). Institutionalizing health technology assessment in ghana: enablers, constraints, and lessons. Health Syst Reform.

[24] Saner H (2019). Digital health implementation: how to overcome the barriers?. Eur J Prev Cardiol.

[25] Guo C, Ashrafian H, Ghafur S, Fontana G, Gardner C, Prime M (2020). Challenges for the evaluation of digital health solutions-A call for innovative evidence generation approaches. NPJ Digit Med.

[26] Opoku D, Busse R, Quentin W (2019). Achieving sustainability and scale-up of mobile health noncommunicable disease interventions in sub-Saharan Africa: Views of policy makers in Ghana. JMIR Mhealth Uhealth.

[27] Williams K, Markwardt S, Kearney SM, Karp JF, Kraemer KL, Park MJ, Freund P, Watson A, Schuster J, Beckjord E (2021). Addressing implementation challenges to digital care delivery for adults with multiple chronic conditions: stakeholder feedback in a randomized controlled trial. JMIR Mhealth Uhealth.

[28] Amoakoh HB, Klipstein-Grobusch K, Grobbee DE, Amoakoh-Coleman M, Oduro-Mensah E, Sarpong C, Frimpong E, Kayode GA, Agyepong IE, Ansah EK (2019). Using mobile health to support clinical decision-making to improve maternal and neonatal health outcomes in Ghana: insights of frontline health worker information needs. JMIR Mhealth Uhealth.

[29] Vayena E, Haeusermann T, Adjekum A, Blasimme A (2018). Digital health: meeting the ethical and policy challenges. Swiss Med Wkly.

[30] Amoakoh HB, Klipstein-Grobusch K, Agyepong IA, Amoakoh-Coleman M, Kayode GA, Reitsma JB, Grobbee DE, Ansah EK (2020). Can an mhealth clinical decision-making support system improve adherence to neonatal healthcare protocols in a low-resource setting?. BMC Pediatr.

[31] Kostkova P (2015). Grand challenges in digital health. Front Public Health.

[32] Rooney L, Rimpiläinen S, Morrison C, Nielsen SL (2018). Review of emerging trends in digital health and care. Digital Health and Care Institute. Glasgow: University of Strathclyde.

