# Supplementary material for: Digital Health as an Enabler of Personalized Medicine in Ghana: Gaps, Opportunities, and Future Directions
Source: J Med Internet Res. 2026 Apr 16;28:e70696. doi: 10.2196/70696 (PMC13133594; doi:10.2196/70696)
Supplement: Multimedia Appendix 1 [file jmir_v28i1e70696_app1.pdf]

Digital Health as an Enabler of Personalised Medicine in Ghana: Gaps, Opportunities, and Future Directions

Godsway Sackey, Babajide Owoyele, Frank Baiden, Stefan Konigorski

Multimedia Appendix 1. Search Strategy and Protocol Details

Protocol Registration

The literature examination informing this Viewpoint followed a pre-registered protocol to enhance transparency and methodological rigor. The protocol is publicly available at the Open Science Framework: https://osf.io/n2krw.

Information Sources and Search Period

We conducted structured database searches covering the period from **January 2000 to April 2024**.

The following sources were searched:

- PubMed
- Scopus
- IEEE Xplore
- Cochrane Library
- Google Scholar
- Directory of Open Access Journals (DOAJ)
- Relevant governmental and institutional websites (e.g., Ghana Ministry of Health, Ghana Health Service, WHO)

The final search update was conducted in April 2024.

Search Strategy

The search strategy was developed iteratively by the research team and combined four thematic concept blocks:

1. **Personalised/Precision Medicine**
2. **Digital Health Technologies**
3. **N-of-1 Trials**
4. **Ghana or Low-Resource Settings**

Search terms were adapted to each database syntax. Boolean operators (“AND”, “OR”) were used to combine terms.

1. Personalised / Precision Medicine Terms

“Bio Individuality”
“Personalised Health Care”
“Personalised Therapeutics”

“Personalised Treatment Plans”
“Personal Genomics”
“Precision Medicine”
“Individualized Therapy”
“Tailored Treatment”
“Genetic Medicine”
“Genomic Medicine”
“Pharmacogenomics”
“Customized Healthcare”
“Stratified Medicine”
“Biomarker-based Treatment”

Combined example:
(“Personalised Medicine” OR “Precision Medicine” OR “Genomic Medicine” OR “Pharmacogenomics” OR “Stratified Medicine”)

2. Digital Health Terms
“Digital Health”
“eHealth”
“mHealth”
“Mobile Health”
“Telehealth”
“Telemedicine”
“Health Informatics”
“Electronic Health Records”
“EHR”
“Digital Therapeutics”
“Health Information Technology”
“Health ICT”
“Remote Monitoring”
“Wearable Devices”
“Health Apps”

Combined example:
(“Digital Health” OR “eHealth” OR “mHealth” OR “Telemedicine” OR “Electronic Health Records” OR “Health Informatics”)

3. N-of-1 Trial Terms
“N-of-1 Trials”
“Single-Patient Trials”
“Single-Subject Research”
“Individualized Trials”
“Personalised Clinical Trials”
“Single-Case Experiments”

4. Geographic / Context Terms
“Ghana”

“Low-Resource Settings”
“Developing Countries”
“Africa”

Example Combined Search String (PubMed)

((“Digital Health” OR “eHealth” OR “mHealth” OR “Telemedicine” OR “Electronic Health Records”)
AND
(“Personalised Medicine” OR “Precision Medicine” OR “Genomic Medicine” OR “Pharmacogenomics”)
AND
(“N-of-1 Trials” OR “Single-Patient Trials” OR “Individualized Trials”))
AND
(Ghana OR “Low-Resource Settings” OR “Developing Countries” OR Africa)

Search strings were modified appropriately for database-specific indexing terms and controlled vocabularies.

Eligibility Criteria

Inclusion Criteria

Studies were included if they:

- Addressed digital health technologies, personalised/precision medicine, or N-of-1 trials
- Focused on Ghana or comparable low-resource settings
- Were empirical studies, systematic reviews, or implementation analyses
- Were published in English
- Were published between January 2000 and April 2024

Exclusion Criteria

Studies were excluded if they:

- Were editorials, commentaries, or opinion-only pieces
- Did not address digital health or personalised approaches
- Focused exclusively on high-income settings without transferable relevance
- Were published prior to 2000
- Were not available in English

Study Selection Process

The selection process involved:

1. Initial screening of titles and abstracts for relevance
2. Full-text review of potentially eligible articles
3. Resolution of discrepancies through discussion among reviewers

Bibliographic records were managed using Zotero for deduplication and metadata cleaning.

Data Extraction

Data were extracted using a structured template capturing:

- Author and year
- Study design
- Setting and population
- Intervention type
- Primary and secondary outcomes
- Relevance to digital health and personalisation
- Contextual applicability to Ghana or similar settings

Extracted data informed the thematic synthesis used to support the interpretive arguments presented in the Viewpoint.

Scope of Identified Literature

The examination identified 40 publications meeting inclusion criteria. The majority focused on mobile health (mHealth) and electronic health systems in Ghana. Explicitly personalised medicine applications were limited, and no empirical N-of-1 trials conducted within Ghana were identified.

Data Availability

The cleaned integrated dataset generated during this literature examination is available from the corresponding author upon reasonable request.
